# Avenanthramide-C ameliorate doxorubicin-induced hepatotoxicity via modulating Akt/GSK-3β and Wnt-4/β-Catenin pathways in male rats

**DOI:** 10.3389/fmolb.2024.1507786

**Published:** 2024-12-02

**Authors:** Maha Abdullah Alwaili, Amal S. Abu-Almakarem, Salwa Aljohani, Sahar Abdulrahman Alkhodair, Maha M. Al-Bazi, Thamir M. Eid, Jehan Alamri, Maysa A. Mobasher, Norah K. Algarzae, Arwa Ishaq A. Khayyat, Luluah Saleh Alshaygy, Karim Samy El-Said

**Affiliations:** ^1^ Department of Biology, College of Science, Princess Nourah bint Abdulrahman University, Riyadh, Saudi Arabia; ^2^ Department of Basic Medical Sciences, Faculty of Applied Medical Sciences, Al-Baha University, Al Bahah, Saudi Arabia; ^3^ Chemistry Department, Faculty of Science, Taibah University, Yanbu, Saudi Arabia; ^4^ Department of Biochemistry, Faculty of Science, King Abdulaziz University, Jeddah, Saudi Arabia; ^5^ Biology Department, Faculty of Science, King Abdulaziz University, Jeddah, Saudi Arabia; ^6^ Department of Pathology, Biochemistry Division, College of Medicine, Jouf University, Sakaka, Saudi Arabia; ^7^ Department of Physiology, College of Medicine, King Saud University, Riyadh, Saudi Arabia; ^8^ Biochemistry Department, Science College, King Saud University, Riyadh, Saudi Arabia; ^9^ Biochemistry Division, Chemistry Department, Faculty of Science, Tanta University, Tanta, Egypt

**Keywords:** avenanthramides, antioxidants, anti-inflammatory, doxorubicin, hepatotoxicity, signaling pathway

## Abstract

**Background:**

Doxorubicin (DOX) drugs used in cancer treatment can cause various adverse effects, including hepatotoxicity. Natural-derived constituents have shown promising effects in alleviating chemotherapy-induced toxicities. This study addressed the effect of Avenanthramides-C (AVN-C) treatment in rats with DOX-indued hepatotoxicity.

**Methods:**

AutoDock Vina was used for the molecular docking investigations. *In silico* toxicity prediction for AVN-C and DOX was performed using the Pro Tox-III server. Four groups of ten male Sprague-Dawley rats were created: Group 1 (Gp1) served as a negative control, Gp2 received an intraperitoneal (i.p.) injection of AVN-C (10 mg/kg), Gp3 received an i.p. dose of DOX (4 mg/kg) weekly for a month, and Gp4 received the same dose of DOX as G3 and AVN-C as G2. Histopathological, molecular, and biochemical analyses were conducted 1 month later.

**Results:**

The study showed that treatment with AVN-C significantly ameliorated DOX-induced hepatotoxicity in rats by restoring biochemical alterations, boosting antioxidant activity, reducing inflammation, and modulating the Akt/GSK-3β and Wnt-4/β-Catenin signaling pathways in male rats.

**Conclusion:**

This study is the first to demonstrate the therapeutic effects of AVN-C therapy on DOX-induced liver damage in male rats. Therefore, AVN-C could have a pronounced palliative effect on the hepatotoxicity caused by DOX treatment. These findings suggest that AVN-C could potentially alleviate the hepatotoxicity associated with DOX-based chemotherapy.

## 1 Introduction

The hepatotoxicity of chemotherapeutic drugs has been noted to require close monitoring by oncologists during treatment, despite growing number of cancer survivors. However, many of these survivors still suffer from symptoms of liver because of their cancer therapy (Mudd and Guddati, 2021). One common first-line treatment for different types of cancer is doxorubicin (DOX), which has been found tocause hepatotoxicity. Several strategies have been developed to prevent liver damage caused by DOX, but additional strategies are needed to protect liver tissues from its harmful effects (Sirwi et al., 2021; Al-Qahtani et al., 2022; El-Said K. S. et al., 2023). Various processes, including apoptosis, inflammation, and oxidative stress, have been proposed to explain the mechanisms behind DOX-induced hepatotoxicity. Therefore, it is crucial to understand the mechanisms that protect liver tissue during DOX administration (Morsy et al., 2023). DOX may induce oxidative stress by blocking the transcription factor Nrf-2, which coordinates cellular redox homeostasis and regulates antioxidant and detoxifying responses (Jakobs et al., 2017). A recent study by Saleh et al. (2022) highlighted the protective role of Omega-3 in DOX-induced hepatotoxicity through Akt/GSK-3β axis modulation in rats. Additionally, Sirwi et al. (2021) demonstrated that Mokko lactone attenuated DOX-induced hepatotoxicity by regulating the Sirt-1/FOXO1/NF-κB axis in rats.

A major redox-sensitive protein kinase, glycogen synthase kinase (GSK)-3β is widely expressed in nearly every cell and plays a role in various intracellular and extracellular processes, including influencing cell development and apoptosis (Martelli et al., 2021). Akt could control the GSK-3β activity, and suppressing GSK-3β could modulate oxidative stress in hepatocytes by targeting Nrf-2 (Jiang et al., 2015). Importantly, GSK-3β signaling seems to be responsible for the apoptosis induction observed upon Akt inhibition (Li et al., 2020). The Akt/GSK-3β signaling pathway is crucial for cell survival and has been linked to the reduction of liver injury through anti-inflammatory and anti-apoptotic effects (Xing et al., 2024). Targeting GSK-3β may be a unique approach to mitigate the toxicity caused by DOX in multiple organs, according to evidence (Niringiyumukiza et al., 2019).

The development, differentiation, and cellular homeostasis of liver tissue rely on the complex Wnt signaling system. The Wnt pathway plays a significant role in liver metabolism, regeneration, and maintaining the normal function of liver (Hu and Monga, 2021). Abnormal regulation of Wnt/β-catenin signaling pathway is associated with various disorders making it a desirable target for disease treatment (Liu et al., 2022). Activation of Wnt/β-catenin signaling pathway is linked to the pathogenesis of liver fibrosis and protective role of natural compounds against liver fibrosis by inhibiting the Wnt/β-catenin pathway has been reported (El-Ashmawy et al., 2020).Phenolic compounds including, anthranilic acid amides, have multiple health promoting qualities, and were known for their antioxidant, anti-inflammatory, and anti-proliferative properties (Toma et al., 2020; Rahman et al., 2021; Mobasher et al., 2024). These anthranilic acid amides, or called avenanthramides (AVNS), are a class of N-cinnamoyl anthranilic acids that oat plants make as phytoalexins (Pretorius and Dubery, 2023). Oats contain AVN in a variety of forms, but AVN-C is the most common and has the strongest antioxidant effect (Perrelli et al., 2018). Additionally, AVNS have been shown to have potent biomedical potential both *in vivo* and *in vitro* through reducing oxidative stress-related disorders and cellular dysfunctions (Perrelli et al., 2018; Wankhede et al., 2023). Previous studies demonstrated the therapeutic effects of AVN-C against lung toxicity in rats, inflammation, and oxidative stress in human skin fibroblasts (Wang and Eskiw, 2019; Altwaijry et al., 2021).). AVNs have also been reported to reduce the risk of colon cancer by targeting apoptosis, which reduces cellular proliferation (Fu et al., 2019). Thisnovel study investigated for the first time the curative role, biochemical pathways, and molecular mechanisms of AVN-C treatment against DOX-induced hepatotoxicity in rats.

## 2 Materials and methods

### 2.1 Chemicals

Avenanthramide-C (AVN-C) was purchased from Sigma-Aldrich (Cat. no. SI0330531, United States) and dissolved in a 10% dimethyl sulfoxide (DMSO) solution in normal saline for *in vivo* study. DOX hydrochloride (Cat. no. D5220, 98%–102% HPLC) was purchased from Sigma-Aldrich (Oakville, ON L6H 6J8), Canada.

### 2.2 Molecular docking analysis

The structures of ligands were retrieved from the PubChem database in SDF format. The 3D structureswere energy-minimized using Avogadro 1.2.0 software with the MMFF94 force field (Hanwell et al., 2012). The structure of the RAC-alpha serine/threonine-protein kinase (Akt-1) in *Rattus norvegicus* (Rat) (UniProt ID: P47196) protein was retrieved from the UniProt database. Glycogen synthase kinase-3 beta (GSK-3β) in *R. norvegicus* (Rat) (UniProt ID: P18266), Wnt protein in *R. norvegicus* (Rat) (UniProt ID: Q9QXQ5), and β-Catenin protein (UniProt ID: Q9WU82) was also obtained. The binding sites for these proteins were predicted based on literature information and validated using the CB-DOCK2. Proteins were prepared for docking using AutoDock Tools 1.5.7 (Morris et al., 2009).

Using AutoDock Vina, molecular docking investigations were conducted (Trott and Olson, 2010) to predict the binding modes and affinities of the compounds with each protein. The grid boxes for docking were centered on the predicted binding sites. The exhaustiveness parameter was set to 8, and the default scoring function was used for the docking calculations. BIOVIA Discovery Studio 2020 (San Diego, CA, United States) was used to visualize and evaluate the docking data. The binding affinities (ΔG values) and intermolecular interactions, including hydrogen bonds and hydrophobic interactions, were analyzed and reported. *In silico* toxicity prediction for AVN-C and DOX was performed using the Pro Tox-III server (Banerjee et al., 2024) by retrieving SMILES codes from PubChem for each compound and adding them to the server.

### 2.3 Rats and experimental design

**FIGURE 1:**
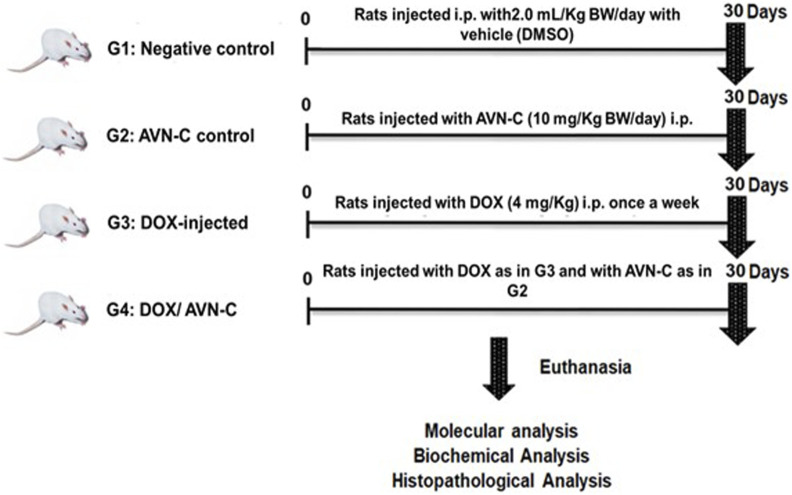
Schematic illustration of experimental design showing the different groups under the study after different treatment settings.

Forty adult male Sprague-Dawley rats (130–150 g, 5–6 weeks of age) were purchased from Helwan University, Egypt. The present study was reported in accordance with the Animal Research: Reporting of *In Vivo* Experiments (ARRIVE) guidelines. The experimental protocol was approved by Tanta University’s Faculty of Science’s Animal Care Committee with approval number (ACUC-SCI-TU-238) in Egypt. All experiments were performed following relevant international and national guidelines and regulations. The rats were equally divided into four groups: G1 was a negative control group injected with the vehicle (DMSO 10% in normal saline), 300 µL/each rat, i.p. daily for a month; G2 was injected with AVN-C (10 mg/kg), 300 µL/each rat, i.p. daily for a month (Umugire et al., 2019); G3 was injected with 4 mg/kg of DOX i.p. once a week for a month (Warpe et al., 2015); and G4 was injected with DOX as in G3 and administered with AVNS-C as in G2. The rats were anesthetized using isoflurane, euthanized, and then blood, serum, and liver tissues were collected for hematological, biochemical, molecular, and histopathological investigations (Figure 1).

### 2.4 Hematological analysis

For hematological analysis, an automated blood cell counter (Sysmex XT-2000i Automated Hematology Analyzer, PU-17) was used. The parameters measured included red blood cell (RBC) count, hemoglobin (Hb) levels, hematocrit (Hct) percentages, mean corpuscular hemoglobin (MCH), mean corpuscular hemoglobin concentration (MCHC), mean corpuscular volume (MCV), platelet count, white blood cell (WBC) count, and differential count for lymphocytes and neutrophils.

### 2.5 Biochemical analysis

Aspartate aminotransferase (AST) (catalog no. AS106145), alanine aminotransferase (ALT) (catalog no. AL103145), alkaline phosphatase (ALP) (catalog no. AP1020), total bilirubin (catalog no. BR1111), and direct bilirubin (catalog no. BR1112) were assessed using colorimetric kits (Spectrum Diagnostics, Egypt). Hepatic levels of malondialdehyde (MDA) (catalog no. MD2529), superoxide dismutase (SOD) (catalog no. SD2521), catalase (CAT) (catalog no. CA2517), and reduced glutathione (GSH) (catalog no. GR2511) were measured by using their kit (Biodiagnostic, Egypt). Protein concentration was measured by the method of Lowry et al. (1951) using bovine serum albumin (BSA) as a standard. Furthermore, rat’s ELISA kits were used for measurement of the inflammatory biomarkers in the liver homogenates of the different groups, including tumor necrosis factor-α (TNF-α) (catalog no. RAB0479), nuclear factor kappa-B (NF-κB) (catalog no. MBS453975), interleukin-6 (IL-6) (catalog no. E-HSEL-R0004), interleukin-1β (IL-1β) (catalog no. E-EL-R0012), and cyclooxygenase-2 (COX-2) (catalog no. MBS266603). Hepatic rats’ phospho-Akt protein levels were evaluated by the rat’s p-Akt (Ser473) ELISA kit (catalog no. MBS775153). Aliquots of liver homogenate were used for determination of GSK-3β (catalog no. MBS7251608), Wnt-4 (catalog no. MBS2885391), and β-Catenin (catalog no. MBS843456) using their rat-specific ELISA kits that were provided by MyBioSource, Inc., San Diego, CA, United States.

### 2.6 Molecular analysis

The mRNA expressions of *Akt-1, GSK-3β, Wnt-4,* and *β-Catenin* genes were evaluated in the liver tissues of the different groups. The primers were prepared using the Primer-Blast program from NCBI (Table 1). Experiments for the detection of all genes, including the housekeeping gene hypoxanthine phosphoribosyl transferase 1 (HPRT), were performed in triplicate. The relative expression of the target genes was estimated (Livak and Schmittgen, 2001).

**TABLE 1:** Forward and reverse primer sequences for RT-PCR.

`Gene	Accession number	Forward sequence (5′–3′)	Reverse sequence (5′–3′)
*Akt*	NM_033230.3	AGGCATCCCTTCCTTACAG	GCCCGAAGTCCGTTATCT
*GSK-3β*	NM_032080.1	GGTGACTTTGACCGGAACGTG	ATTGAAGGGACAGGTGAACAGG
*Wnt-4*	NM_053402.2	GCCACGCACTAAAGGAGAAG	TCATCCGTATGTGGCTTGAA
*β-Catenin*	NM_001431665.1	CAGATCCCATCCACGCAGTT	TCTGTGACGGTTCAGCCAAG
*GAPDH*	NM_017008.4	CCGCATCTTCTTGTGCAGTG	GAGAAGGCAGCCCTGGTAAC

*Akt-1*, RAC-alpha serine/threonine-protein kinase; *GSK-3β*, Glycogen synthase kinase; *Wnt-4*, Wnt family member 4; *GAPDH*, Glyceraldehyde-3-phosphate dehydrogenase*.*

### 2.7 Histopathological investigations

Liver tissues were sectioned at 5 μm, embedded in paraffin wax, washed in xylene, and then sliced and fixed in 10% buffered formalin. Hematoxylin and eosin (H&E) staining was applied to the sections, which were then observed under an Olympus CX31 light microscope and photographed with a digital camera (Olympus Camedia 5060, Japan) (Bancroft and Gamble, 2008). Hepatic damage was analyzed based on the severity percentage of hepatic tissue using the following scales (0–4): 0 indicated normal tissue, 1 indicated <25% damage, 2 indicated 26%–50% damage, 3 indicated 51%–75% hepatic damage, and 4 indicated >75% (Orabi et al., 2020).

### 2.8 Statistical analysis

A one-way analysis of variance (ANOVA) was conducted to assess significant variations. The software GraphPad Prism (San Diego, CA, United States) was utilized for data analysis. Tukey’s test was used for multiple comparisons, with statistical significance at *p* < 0.05.

## 3 Results

### 3.1 Avenanthramide-C and doxorubicin interactions with target proteins (Akt-1, GSK-3β, Wnt-4, and β-Catenin) by molecular docking analysis

**FIGURE 2:**
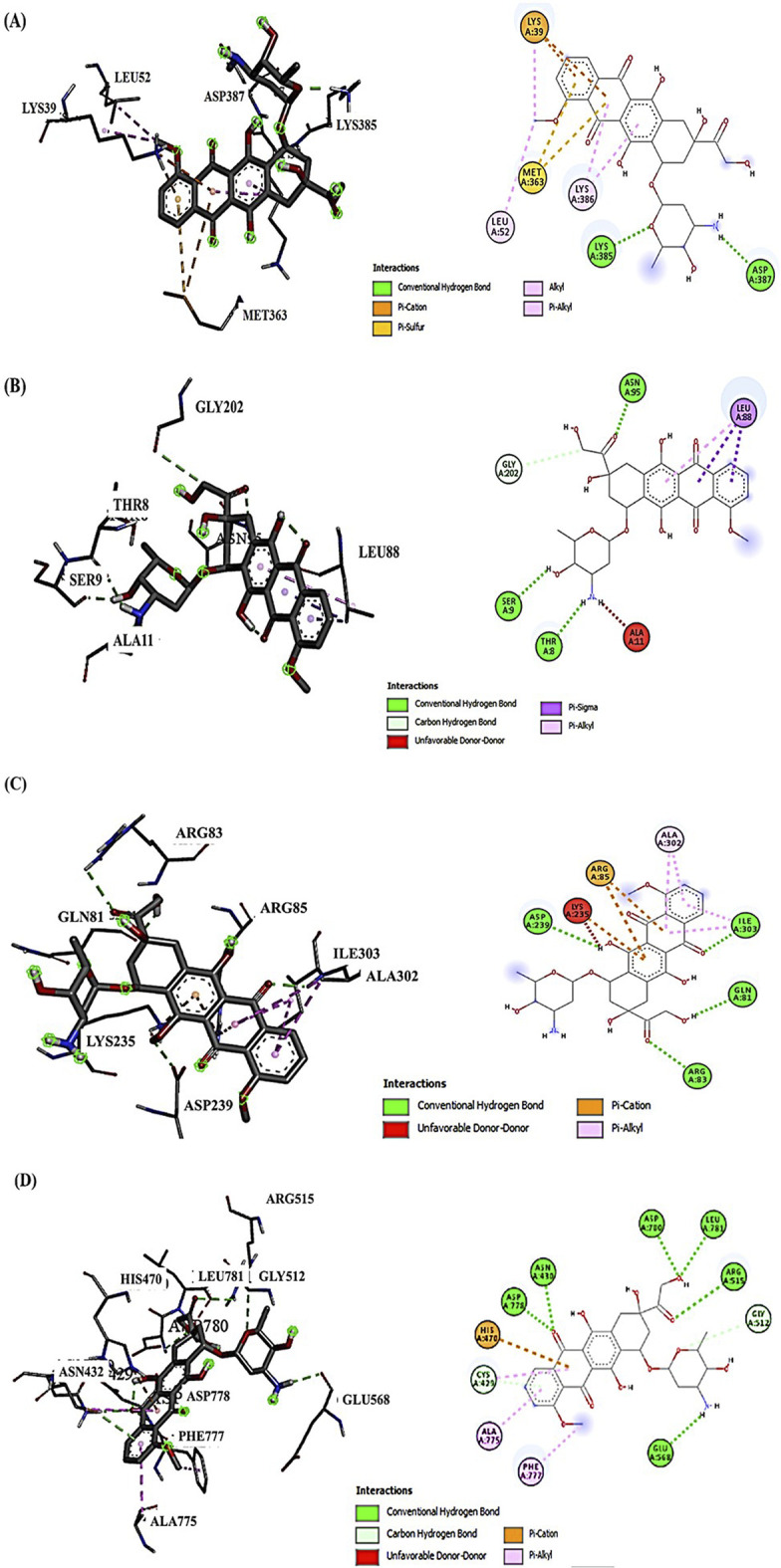
**(A)** Molecular docking analysis shows the interaction of DOX with Akt-1, the conventional H-bond (LYS385 and ASP387); the electrostatic interactions via Pi-cation (LYS39); the hydrophobic interactions (LYS39, LEU52, and LYS386). **(B)** DOX interaction with GSK-3β, conventional H-bond (ASN95, THR8, SER9, and GLY202); carbon H- Bond (GLY202); the hydrophobic interactions (LEU88). (**C)** DOX interaction with Wnt-4; conventional H-bond (ARG83, ILE303, GLN81, and ASP239); Electrostatic Pi-cation interactions (ARG85 and LYS235); Hydrophobic Pi-Alkyl interaction (ALA302, ALA302, and ILE303). **(D)** DOX interaction with β-Catenin, conventional H-bond (ARG515, LEU781, GLU568, and ASN432); carbon H- Bond (CYC429); Pi-cation interaction (HIS470); Pi-Alkyl interaction (ALA775 and PHE777).

**FIGURE 3:**
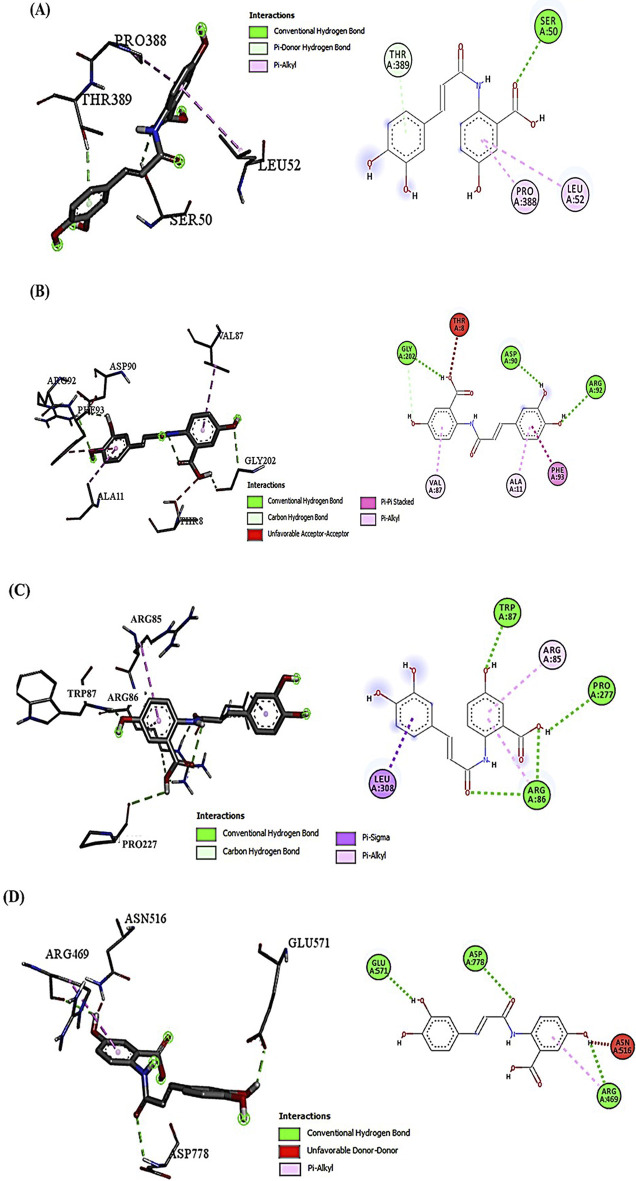
**(A)** Molecular docking analysis shows the interaction of AVN-C with Akt-1, the conventional H-bond (SER50 and THR389); the hydrophobic Pi-Alkyl interactions (LEU52 and PRO388). **(B)** AVN-C interaction with GSK-3β, conventional H-bond (ARG92, ASP90, and GLY202); the hydrophobic Pi-Pi Stacked interactions (PHE93); the hydrophobic Pi-Alkyl interactions (VAL87 and ALA11). **(C)** AVN-C interaction with Wnt-4; conventional H-bond (ARG86, TRP87, and PRO277); Hydrophobic Pi-Sigma interaction (LEU308); Hydrophobic Pi-Alkyl interaction (ARG85). **(D)** AVN-C interaction with β-Catenin, conventional H-bond (GLU571, ASP778, and ARG469); Unfavorable donor-donor bond (ASN516).

Examining the docking results, it has been observed that DOX generally exhibits stronger binding affinities across the range of target proteins compared to AVN-C. This trend is particularly notable for proteins such as β-catenin (−9.1 kcal/mol for DOX vs. −7.6 kcal/mol for AVN-C) (Figures 2, 3). These differences in binding energies suggest that DOX may have a more potent effect on Wnt/β-catenin cellular pathways, which could contribute to both its therapeutic efficacy and potential adverse effects. Delving deeper into the protein-ligand interactions, both compounds have been found to be engaged in a complex network of hydrogen bonds, hydrophobic interactions, and in some cases, electrostatic interactions with target proteins (Akt-1, GSK-3β, Wnt-4). The interaction between AVN-C revealed multiple hydrogen bonds, including a conventional hydrogen bond between SER50 and the ligand, as well as pi-alkyl interactions with LEU52 and PRO388 (Figure 3). In contrast, DOX’s interaction with Akt shows a more extensive network of bonds, including pi-cation interactions with LYS39 and multiple hydrophobic interactions. This more comprehensive binding profile could explain DOX’s higher binding affinity and its known effects on cell cycle regulation and apoptosis through the Akt pathway. The interactions with other proteins, such as GSK-3β, and Wnt follow similar patterns. DOX consistently forms more numerous and varied interactions, which likely contribute to its broader impact on cellular functions (Figures 2, 3). AVN-C is not predicted to exhibit toxicities including, hepatotoxicity, mutagenicity, and cytotoxicity, while DOX is flagged as toxic for these endpoints. This correlates with the stronger binding of DOX to proteins like β-catenin, which are involved in cell signaling and transport processes that could influence these toxicity outcomes (Table 2).

**TABLE 2:** *In silico* toxicity for AVN-C and DOX.

Classification	Target	AVN-C		DOX	
		Prediction	Probability	Prediction	Probability
Organ toxicity	Hepatotoxicity	Inactive	0.59	Active	0.86
Organ toxicity	Neurotoxicity	Inactive	0.7	Active	0.74
Organ toxicity	Nephrotoxicity	Active	0.64	Active	0.8
Organ toxicity	Respiratory toxicity	Active	0.53	Active	0.91
Organ toxicity	Cardiotoxicity	Inactive	0.96	Active	0.64
Toxicity end points	Carcinogenicity	Inactive	0.52	Active	0.9
Toxicity end points	Immunotoxicity	Inactive	0.89	Active	0.99
Toxicity end points	Mutagenicity	Inactive	0.76	Active	0.98
Toxicity end points	Cytotoxicity	Inactive	0.7	Active	0.94
Toxicity end points	BBB-barrier	Inactive	0.63	Inactive	1
Toxicity end points	Ecotoxicity	Inactive	0.69	Inactive	0.58
Toxicity end points	Nutritional toxicity	Inactive	0.73	Inactive	0.69
Tox21-signalling pathways	Androgen receptor	Inactive	0.95	Inactive	0.99
Tox21-signalling pathways	Androgen receptor ligand binding domain	Inactive	0.99	Inactive	0.55
Tox21-signalling pathways	Aromatase	Inactive	0.95	Active	0.52
Tox21-signalling pathways	Peroxisome proliferator activated receptor gamma (PPAR-Gamma)	Inactive	0.98	Inactive	0.97
Tox21-Stress response pathways	Nrf-2/ARE	Inactive	0.92	Inactive	0.98
Tox21-Stress response pathways	Heat shock factor response element (HSE)	Inactive	0.92	Inactive	0.98
Tox21-Stress response pathways	Tumor supressor protein (p53)	Inactive	0.8	Active	0.52
Molecular Initiating Events	Thyroid hormone receptor beta	Inactive	0.61	Inactive	0.78
Molecular Initiating Events	Transtyretrin (TTR)	Inactive	0.56	Inactive	0.97
Molecular Initiating Events	GABA receptor (GABAR)	Inactive	0.84	Inactive	0.96
Molecular Initiating Events	Kainate receptor	Inactive	0.99	Inactive	0.99
Molecular Initiating Events	NADH-quinone oxidoreductase	Inactive	0.93	Inactive	0.97
Metabolism	Cytochrome CYP1A2	Inactive	0.77	Inactive	0.99
Metabolism	Cytochrome CYP2C19	Inactive	0.95	Inactive	0.97
Metabolism	Cytochrome CYP2C9	Inactive	0.52	Inactive	0.73
Metabolism	Cytochrome CYP2D6	Inactive	0.9	Inactive	0.92
Metabolism	Cytochrome CYP3A4	Inactive	0.82	Inactive	0.98
Metabolism	Cytochrome CYP2E1	Inactive	1	Inactive	0.99

### 3.2 Effects of the treatment with DOX/AVN-C on the percentage of rat’s body weight changes

**FIGURE 4:**
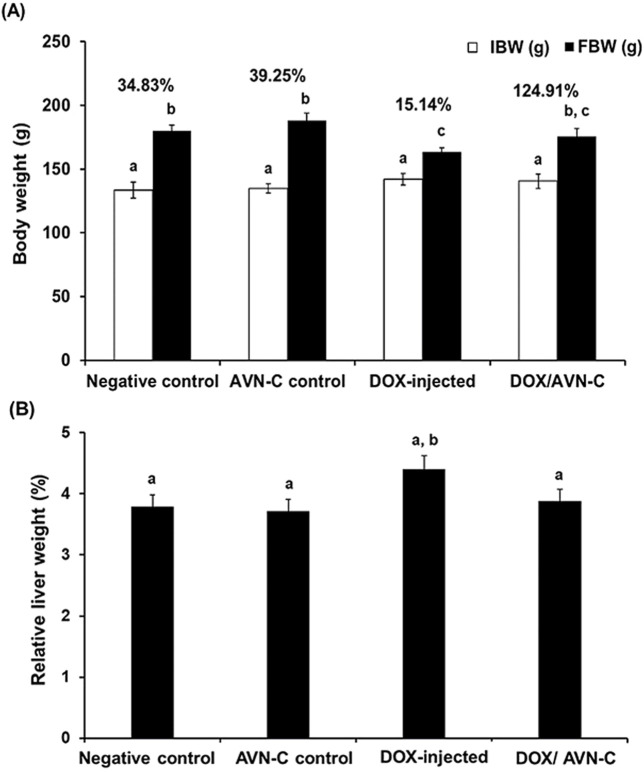
Changes of the body weight (g) **(A)** and the relative liver weight (%) **(B)** in the different groups. The values were represented as means ± S.D. (*n* = 10). IBW: initial body weight; FBW: final body weight; AVN-C: avenanthramide-C; DOX: doxorubicin. Means that do not share letters indicated significant differences (*p < 0.05).

The percentage of body weight changes (% b. wt) in the group injected with DOX were significantly decreased (*p* < 0.05) to 15.14% ± 1.87 when compared to the negative control and AVN-C control groups (34.83% ± 2.65% and 39.25% ± 3.07, respectively). Combining DOX and AVN-C treatment resulted in a significant increase in % b. wt changes to 24.91% ± 2.55 compared to the DOX-administered group alone (Figure 4A). Additionally, the relative liver weight of the DOX-treated group was higher than that of the other experimental groups (Figure 4B).

### 3.3 Effect of DOX/AVN-C treatment on the hematological parameters of rats

The results indicated no significant alterations in the hematological parameters between the negative control and AVN-C control groups. Rats co-treated with DOX + AVN-C exhibited a significant improvement (*p* < 0.001) in most hematological parameters compared to rats treated with DOX alone. These results were supported by increases in RBCs count, Hb concentration, Hct%, MCH, MCHC, MCV values, total platelets, and WBC counts. However, significant reductions in RBCs, platelets, and WBCs count as well as significant increases (*p* < 0.001) or (*p* < 0.0001) in lymphocyte counts, were observed in DOX-injected rats compared to control groups (Tables 3, 4).

**TABLE 3:** Effect of AVN-C treatment on the hematological parameter’s alterations induced by DOX in rats.

Groups	RBCs (x10^6^/µl)	Hb (g/dL)	MCH (pg)	MCHC (g/dL)	MCV (fL)
Control	8.95 ± 0.45^a^	13.29 ± 0.78^a^	18.43 ± 0.45^a^	27.87 ± 1.25^a^	75.63 ± 2.37^a^
AVN-C	9.16 ± 0.67^a^	13.56 ± 0.69^a^	18.67 ± 0.39^a^	29.15 ± 1.47^a^	78.29 ± 2.86^a^
DOX	5.21 ± 0.39^c^	9.01 ± 0.84^b^	11.38 ± 0.56^b^	16.95 ± 1.67^b^	59.79 ± 2.53^b^
DOX + AVN-C	7.32 ± 0.59^e^	11.65 ± 0.47^a^	14.23 ± 0.69^c^	23.24 ± 1.83^e^	68.27 ± 2.68^a,b^

The values were represented as means ± S.D. (*n* = 10). RBC, red blood cell; Hb, hemoglobin; Hct, hematocrit; MCH, mean corpuscular hemoglobin; MCHC, mean corpuscular hemoglobin concentration; MCV, mean corpuscular volume; AVN-C, avenanthramide-C; DOX, doxorubicin. Means that do not share letters in each column indicated significant differences.

**TABLE 4:** Effect of AVN-C treatment on the hematological parameter’s alterations induced by DOX in rats.

Groups	Hct (%)	Platelets (x10^3^/mm^3^)	WBCs (x10^3^/µl)	Lymphocytes (x10^3^/µl)	Neutrophiles (x10^3^/µl)
Control	44.63 ± 1.02^a^	734 ± 5.38^b^	8.87 ± 0.47^a^	24.31 ± 2.18^c^	37.42 ± 2.36^b^
AVN-C	46.75 ± 0.98^a^	752 ± 4.96^b^	9.02 ± 0.56^a^	22.65 ± 2.39^c^	38.97 ± 2.52^b^
DOX	29.37 ± 0.33^b^	486 ± 3.78^c^	4.98 ± 0.38^c^	39.82 ± 2.44^b^	21.67 ± 2.54^c^
DOX + AVN-C	36.18 ± 1.23^a,b^	603 ± 4.67^a^	6.74 ± 0.45^a,c^	28.78 ± 1.95^b,c^	28.49 ± 2.28^b,c^

The values were represented as means ± S.D. (*n* = 10). WBC, white blood cell; AVN-C, avenanthramide-C; DOX, doxorubicin. Means that do not share letters in each column indicated significant differences.

### 3.4 Treatment with AVN-C recovers hepatic function markers in DOX-injected rats

Assessing liver function, respective enzymes, and total protein contents are crucial for quantifying liver damage, evaluating the ameliorative efficacy of AVN-C against DOX-induced hepatotoxicity. The current study showed that the DOX-administered group demonstrated significant increase (*p* < 0.05) in the liver transaminase (ALT and AST) levels to 68.52 ± 1.95 and 98.58 ± 2.47 U/L, respectively compared to the negative control group (27.32 ± 0.59 and 41.38 ± 1.65) or the AVN-C control group (24.69 ± 0.68 and 39.67 ± 1.34). However, treating DOX-injected rats with AVN-C resulted in a considerable reduction in hepatic transaminase levels to 43.21 ± 2.15 and 63.21 ± 2.17 U/L, respectively (Table 5). Additionally, the DOX-challenged group showed a significant increase (*p* < 0.05) in ALP levels compared to the negative control and AVN-C control groups (468.47 ± 6.65 U/L versus 246.88 ± 3.95 U/L and 249.31 ± 5.16 U/L). Treatment with DOX + AVN-C led to a significant decrease in serum ALP levels (336.85 ± 5.93 U/L) compared to the DOX-injected group alone (468.47 ± 6.65 U/L). Furthermore, GGT activities were significantly increased (*p* < 0.05) by DOX injection in rats; however, AVN-C treatment decreased those levels. On the other hand, the total hepatic protein contents were significantly decreased in the DOX-challenged group and AVN-C treatment restored those levels in the rats’ livers (Table 5).

**TABLE 5:** Effect of AVN-C treatment on serum liver function parameters’ alterations induced by DOX in rats.

Groups	ALT (U/L)	AST (U/L)	ALP (U/L)	GGT (U/L)	T.P. (mg/dL)
Control	27.32 ± 0.59^a^	41.38 ± 1.65^c^	246.88 ± 3.95^a^	6.78 ± 0.75^b^	7.54 ± 0.39^d^
AVN-C	24.69 ± 0.68^a^	39.67 ± 1.34^c^	249.31 ± 5.16^a^	5.64 ± 0.81^b^	7.95 ± 0.47^d^
DOX	68.52 ± 1.95^c^	98.58 ± 2.47^b^	468.47 ± 6.65^b^	17.43 ± 1.85^a^	3.68 ± 0.24^a^
DOX + AVN-C	43.21 ± 2.15^d^	63.21 ± 2.17^e^	336.85 ± 5.93^c^	9.94 ± 0.98^b,c^	5.23 ± 0.69^d,e^

The values were represented as mean ± S.D. (*n* = 10). ALT, alanine transaminase; AST, aspartate transaminase; ALP, alkaline phosphatase; GGT, gamma-glutamyl transferase; T.P, total proteins; AVN-C, avenanthramide-C; DOX, doxorubicin. Means that do not share letters in each column indicated significant differences.

### 3.5 Treatment with AVN-C mitigates hepatic oxidative stress induced by DOX in rats

**FIGURE 5:**
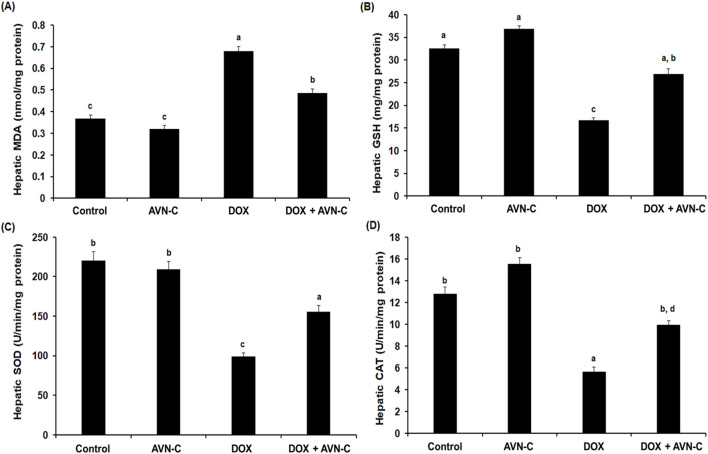
Hepatic levels of malondialdehyde (MDA) **(A)**, reduced glutathione (GSH) **(B)**, superoxide dismutase (SOD) **(C)**, and catalase (CAT) **(D)** in the different groups. AVN-C: avenanthramide-C; DOX: doxorubicin. The values were represented as means ± S.D. (*n* = 10). Means that do not share letters indicated significant differences (* *p <* 0.05).

When compared to the control groups, the DOX-exposed group had a 2.2-fold increase in the hepatic levels of MDA. The rats that were co-treated with DOX + AVN-C demonstrated a significant decrease (*p* < 0.05) in the MDA level compared to the DOX-injected group (0.487 ± 0.019 nmol/mg protein versus 0.679 ± 0.023 nmol/mg protein) (Figure 5A). In contrast, the GSH level was significantly decreased in the DOX-injected group by −2.3 folds compared to the control groups. The rats that were injected with DOX and treated with AVN-C showed a significant increase in the hepatic GSH content compared to the DOX-injected group alone (26.93 ± 1.18 mg/mg protein versus 16.67 ± 0.59 mg/mg protein) (Figure 5B). The hepatic SOD and CAT activities were significantly decreased (*p* < 0.05) upon DOX injection in rats; however, the concomitant treatment with DOX/AVN-C led to a significant restoration of SOD and CAT activities (Figures 5C, D).

### 3.6 AVN-C treatment showed ant-inflammatory properties in DOX-injected rats

**FIGURE 6:**
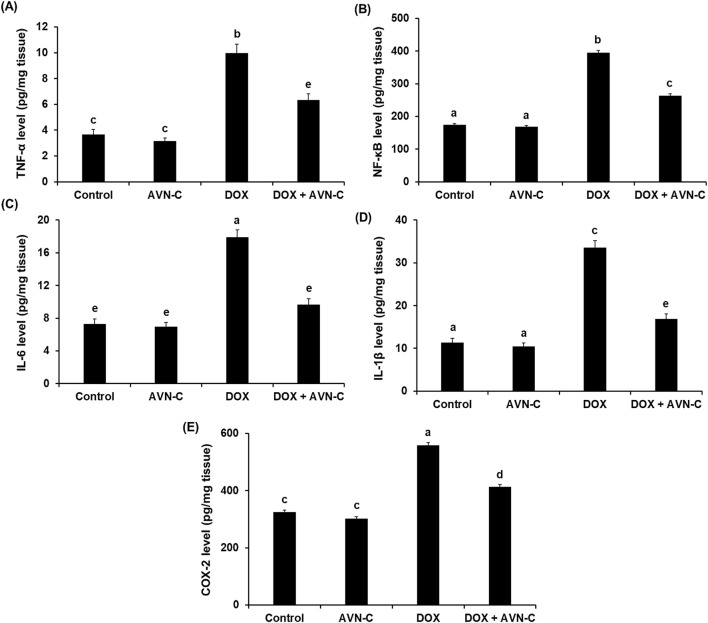
Hepatic levels of tumor necrosis factor alpha (TNF-α) **(A)**, nuclear factor kappa-B (NF-κB) **(B)**, interleukin-6 (IL-6) **(C)**, interleukin-1β (IL-1β**) (D)**, and cyclooxygenase-2 (COX-2) **(E)** in the different groups. AVN-C: avenanthramide-C; DOX: doxorubicin. The values were represented as means ± S.D. (*n* = 10). Means that do not share letters indicated significant differences (* *p <* 0.01).

The results showed that injection of DOX in rats resulted in a significant increase (*p* < 0.01) in the levels of inflammatory biomarkers, including TNF-α, NF-κB, IL-6, IL-1β, and COX-2 (9.97 ± 0.84, 394.65 ± 7.05, 17.93 ± 1.03, 33.57 ± 1.93, and 558.68 ± 6.24 pg/mg tissue, respectively) when compared with the control groups (Figure 6). However, these inflammatory cytokines were significantly reduced (*p* < 0.01) by treating the DOX-challenged rats with AVN-C (6.33 ± 0.55, 263.84 ± 4.77, 9.66 ± 0.71, 16.88 ± 1.14, and 413.67 ± 5.44 pg/mg tissue, respectively) (Figure 6).

### 3.7 Treatment with AVN-C modulates Akt/GSK-3β and Wnt-4/β-Catenin pathways in DOX-injected rats

**FIGURE 7:**
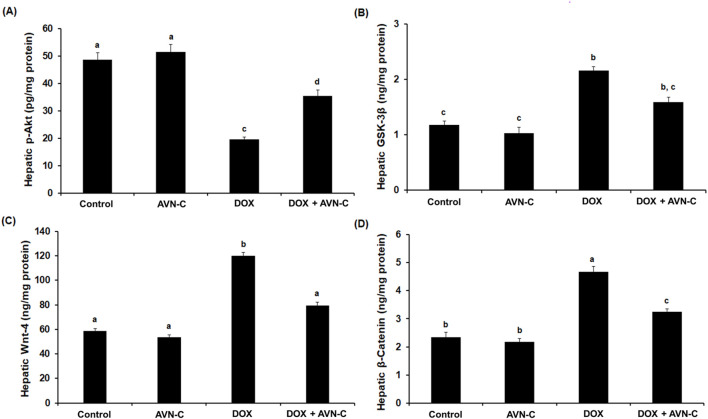
Hepatic levels of RAC-alpha serine/threonine-protein kinase (Akt) **(A)**, Glycogen synthase kinase (GSK-3β) **(B)**, Wnt family member 4 (Wnt-4) **(C)**, and β-Catenin **(D)** in the different groups. AVN-C: avenanthramide-C; DOX: doxorubicin. The values were represented as means ± S.D. (*n* = 10). Means that do not share letters indicated significant differences (* *p <* 0.05).

**FIGURE 8:**
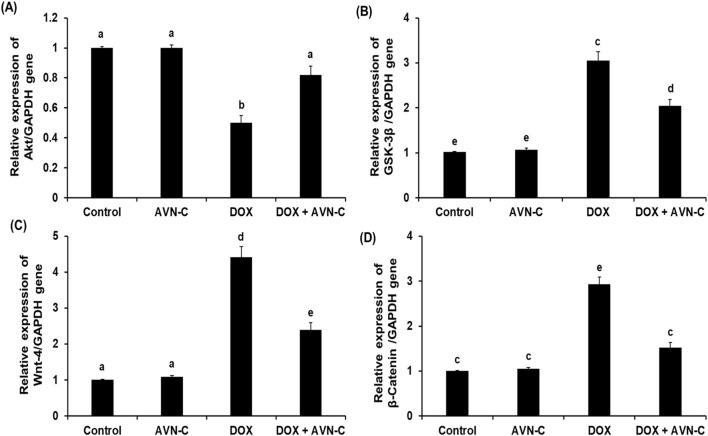
Relative mRNA expression levels of the hepatic RAC-alpha serine/threonine-protein kinase (Akt) **(A)**, Glycogen synthase kinase (GSK-3β) **(B)**, Wnt family member 4 (Wnt-4) **(C)**, and β-Catenin **(D)** in the different groups. AVN-C: avenanthramide-C; DOX: doxorubicin. The values were represented as means ± S.D. (n = 10). Means that do not share letters indicated significant differences (*p < 0.01).

As shown in Figure 7, the intraperitoneal injection of 4 mg/kg DOX once a week for a month in rats led to a significant reduction (*p* < 0.05) in the hepatic pAkt by approximately −2.5 folds (19.59 ± 0.85 Pg/mg protein) versus the negative control (48.59 ± 2.56 Pg/mg protein) and the AVN-C control group (51.39 ± 2.68 Pg/mg protein). This reduction was restored by the treatment with AVN-C (35.44 ± 2.14 pg/mg protein) (Figure 7A). Contrary, intraperitoneal injection of DOX led to a notable increment in hepatic GSK-3β levels to 2.16 ± 0.074 ng/mg protein compared to the negative control (1.18 ± 0.068 ng/mg protein) and AVN-C control groups (1.03 ± 0.105 ng/mg protein). Treatment with AVN-C (10 mg/kg) i.p. daily for a month significantly inhibited (*p* < 0.05) hepatic GSK-3β levels to 1.59 ± 0.094 ng/mg protein, representing a 36% decrease when compared to DOX-treated rats (Figure 7B). Similarly, DOX-induced hepatotoxicity in rats showed significant increases (*p* < 0.05) in Wnt-4 and β-Catenin by 2.2 and 2.1 folds, respectively, when compared to the negative and AVN-C control groups. However, the rats administered with DOX- and treated with AVN-C demonstrated a significant reduction (*p* < 0.05) in hepatic levels of Wnt-4 and β-Catenin, as their levels reaching 79.47 ± 2.75 and 3.25 ± 0.098 ng/mg protein, respectively (Figure 7). Moreover, the gene expression analysis of the *Akt* gene in the DOX-treated group showed significant downregulation (*p* < 0.01) using *GAPDH* gene as a housekeeping genecompared to control groups. However, the DOX/AVN-C treated group showed significant restoration of the Akt gene. Conversely, the *GSK-3β*, *Wnt-4*, and *β-Catenin* genes were significantly downregulated (*p* < 0.01) in the DOX + AVN-C co-treated group compared to the DOX-treated group alone (Figure 8).

### 3.8 AVN-C treatment restores liver histopathological alterations in DOX-injected rats

**FIGURE 9:**
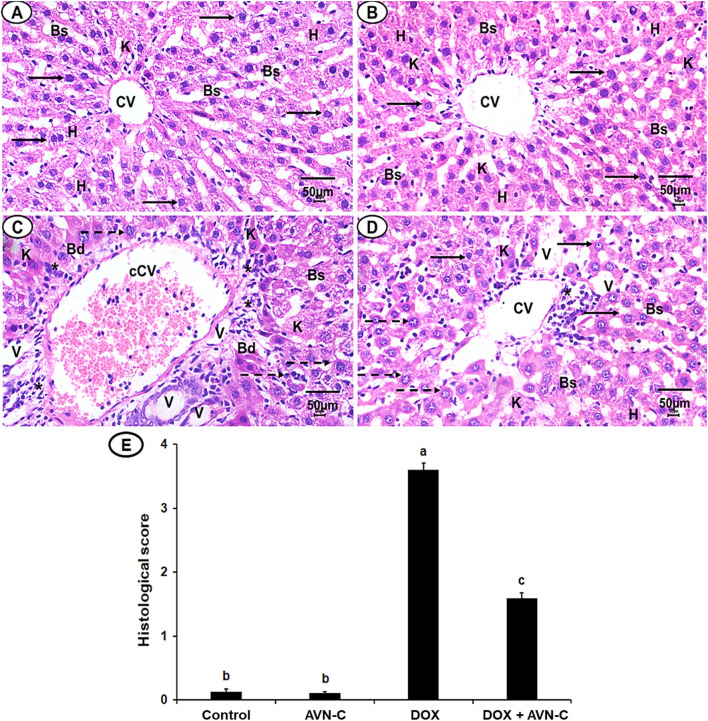
**(A)** A photomicrograph of the liver section from the negative control group shows organized hepatic architecture, centered nucleus (arrows), central veins (CV), normal hepatocytes (h), normal blood sinusoids (Bs) and Kupffer cells (k). **(B)** Liver section of AVN-C control group shows mostly normal hepatocytes, nucleus with normal Bs and (k) **(C)** Liver section of DOX-injected group demonstrated disorganization hepatic structures, congested CV (cCV), cellular infiltrations (*), vacuolated cytoplasm (V), pyknotic nuclei (dotted arrows). **(D)** Liver section of the DOX + AVN-C-treated group demonstrated the improvement in the hepatic organization, fewer congestion, binucleated hepatocytes, and cellular infiltrations (H&E × 400, scale bar = 50 μm). **(E)** Liver sections histological scores in the different groups.

Histopathological analysis revealed normal hepatocyte architecture, a central hepatic vein with centrally located nuclei in the liver sections of the negative control and AVN-C-control groups, representing pathological scores of 0.13 ± 0.04 and 0.11 ± 0.02, respectively (Figures 9A, B, E). The liver section of the DOX-injected group demonstrated severe hepatocyte degeneration, with an acutely dilated central vein, cellular swelling, and nuclear changes. The semi-quantitative analysis showed high pathological scores recorded as 3.60 ± 0.11 (Figures 9C, E). The liver section of the DOX + AVN-C-administered group displayed a significant improvement in hepatic structure and less congestion that represented pathological scores of 1.50 ± 0.09 (Figures 9D, E).

## 4 Discussion

Cancer patient survival rates have gradually increased due to advancements in chemotherapy and treatment plans (Bluethmann et al., 2016). The traditional usage of DOX has been limited due to its adverse effects on various organs, including hepatotoxicity (Prasanna et al., 2020). The use of natural herbal constituents against hepatotoxicity mediated by DOX has been reviewed (Mahmoudi et al., 2023). The phenolic compounds (AVNs) exhibit high antioxidant properties, their efficiency against chemotherapy-induced toxicity has been reported in experimental animals (Umugire et al., 2022). A previous study reported that AVNs could decrease liver dysfunctions induced by cisplatin, in mice (El-Said K. et al., 2023). Therefore, this study aimed to evaluate the efficacy of AVN-C treatment versus hepatotoxicity in rats promoted by DOX through *in silico* studies, hematological, biochemical, molecular, and histopathological investigations. Furthermore, this study explored the mechanisms of AVN-C action on DOX-induced hepatotoxicity through antioxidant, anti-inflammatory, and modulation of Akt/GSK-3β and Wnt-4/β-Catenin pathways in rats.

Molecular docking studies revealed insights into the binding affinities and interaction patterns of AVN-C and DOX with key proteins involved in various cellular processes. Further investigation of protein-ligand interactions showed that both substances interacted with target proteins (Akt-1, GSK-3β, Wnt-4) through hydrogen bonds, hydrophobic contacts, and occasionally electrostatic interactions. These interactions suggest that AVN-C may modulate Akt signaling, potentially influencing cell survival and proliferation pathways. *In Silico* toxicity predictions provided insights into the safety profiles of AVN-C and DOX, withAVN-C predicted to be less toxic across several endpoints compared to DOX. This differential interaction helps explain the distinct effects observed when each compound is administered. Inhibition of p-Akt led to activation of GSK-3β, *in silico* studies revealed that the binding of DOX with GSK-3β does not suppress the vigorous activation of GSK-3β proteins, this could be due to the compensatory mechanisms of hepatocytes to overexpress GSK-3β for oxidative stress, inflammation, and apoptosis inductions, while co-treatment with DOX and AVN-C could reduce GSK-3β protein expression levels. The suppression of P-Akt levels by DOX in the *in-vivo* studies aligns with its strong interaction with Akt, indicating that DOX may inhibit Akt phosphorylation, leading to reduced cell survival signaling. This can contribute to increased apoptosis and liver damage. A previous study of Saleh et al. (2022) reported that suppression of hepatic GSK-3β levels upon treatment with omega-3 as compared to the DOX-treated group as well as restoration in hepatic levels of p-Akt, suggesting the GSK-3β inhibitor’s protective properties against DOX-induced oxidative stress. The upregulation of Wnt-4 and β-catenin expressions suggested also that DOX alters the balance of these pathways, promoting compensation processes in liver cells to overexpress these proteins that can lead to hepatocytes dysfunctions or death. Co-treatment with DOX + AVN-C led to an increase in P-Akt levels and the decrease in GSK-3β, Wnt-4, and β-catenin expressions suggest a protective effect of AVN-C. *In Silico* studies supports this by showing that AVN-C, having lower binding affinity, may act as a milder modulator of these pathways, but when co-treated and reacted with DOX *in vivo* counteracting the deleterious effects of DOX on the target proteins. These abilities of AVN-C to shift the expression patterns towards those observed in healthy liver function can be attributed to its potential to modulate the effects of DOX on these proteins.

The interaction of DOX with multiple targets points to a complex network of signaling pathways that are affected by its administration causes signaling pathway crosstalk. The *in silico* findings provide a mechanistic basis for understanding how changes in protein interactions could lead to the observed *in vivo* outcomes, particularly regarding the interplay between survival and apoptotic signals. The results suggest that AVN-C might mitigate some of the harmful effects of DOX by partially restoring the signaling balance, enhancing cell survival signals while dampening pro-apoptotic signals.

The hepatotoxicity prediction is particularly interesting, given DOX’s known clinical hepatotoxic effects and supports the idea that its stronger interactions with key proteins may contribute to this side effect. However, AVN-C is not entirely devoid of potential toxicity, albeit with lower probabilities compared to other endpoints. This suggests that while AVN-C may have a more favorable overall safety profile,. Interestingly, both compounds are predicted to be inactive for most Tox21 nuclear receptor signaling pathways and stress response pathways, suggesting that their mechanisms of action and potential toxicity may not be mediated through these specific pathways. The integration of molecular docking results with toxicity predictions paints a nuanced understanding of how these compounds might behave in biological systems. Doxorubicin’s stronger binding affinities across multiple proteins correlate with its predicted higher toxicity and known clinical effects. Its interactions with proteins like Akt, GSK-3β, and β-catenin suggest a multi-faceted impact on cell signaling, explaining both its therapeutic efficacy for hepatotoxicity and adverse effects induced by DOX.

Treatment with DOX led significant body weight loss and a substantial decrease in food intake, decreases in fat, skeletal muscle mass, and fatigue (de Lima Junior et al., 2016). In accordance with our study, DOX administration in rats caused significant body weight loss; this could be due to the toxic effects of DOX on metabolism and vital organs. The effect of DOX on the body weight of rats can be attributed to decreased appetite, reduced feed intake, disruption of basal metabolism, and inhibition of protein synthesis (El-Said K. S. et al., 2023). However, the treatment of DOX-injected rats with AVN-C resulted in a significant improvement in the % BW; this finding agreed with previous reports on the impacts of herbal products on the improvement of body weight loss induced by DOX injection (Koss-Mikołajczyk et al., 2021; Saleh et al., 2022; Alherz et al., 2023). Additionally, in response to liver damage caused by DOX, there were inflammatory responses and compensatory mechanisms that promote liver regeneration leading to an increase in liver weight. This increase in liver weight could also be attributed to cellular hypertrophy, surviving hepatocytes may increase in size due to hepatocytes vacuolation, degeneration of hepatocyte cords, bile duct hyperplasia and focal necrosis, as they attempt to compensate for lost functionality, leading to increased liver mass despite reduced protein content (Prasanna et al., 2020; Xu et al., 2022). Also, this could be due to altered protein turnover and protein metabolism might shift; although total protein content decreased, the liver might still be undergoing processes like protein degradation at a higher rate due to oxidative protein damage. While protein content decreased due to damage, the liver may still activate pathways to maintain its mass and function, such as increased glycogen storage or lipid accumulation. Additionally, the increase in liver weight after DOX treatment could be due to the accumulation of extracellular matrix components, which can alter liver structures (Jurj et al., 2022; Gedikli et al., 2023).

It has been reported that DOX treatment induced bone marrow and spleen immunosuppression in rats (Shaldoum et al., 2021). In this study, the outcomes reported that DOX injection in rats led to significant alterations in the hematological parameters, including RBCs, Hb, WBCs, and platelets. These reductions in the hematological parameters from DOX treatment could be due to damage in the hematopoietic system or the increased permeability of the cell membrane, which in turn caused osmotic swelling and erythrocyte hemolysis and the occurrence of systemic inflammation (Kameo et al., 2021). Additionally, hypoxia may potentially have been caused by the death of the mature cell, an increase in plasma volume, or a decrease in hemoglobin’s affinity for oxygen, which resulted in less oxygen being transported from the lungs to the blood and less oxygen being released from oxyhemoglobin to the tissues (Jagetia et al., 2006). Treating DOX-injected rats with AVN-C showed significant improvement in the previously mentioned hematological parameters as evidenced by restoration of RBCs, WBCs, and platelet counts and improvement of bone marrow thrombopoietin, megakaryocytopoiesis, and scavenging free radical-induced damage in cells (Fathy et al., 2018; El-Said et al., 2022). These results were in line with previous studies reporting the beneficial effects of natural constituents on improving the hematological parameter alterations that were induced by DOX in experimental animals (Afsar et al., 2017; Espírito Santo et al., 2023).

Our outcomes uncovered that DOX induced liver dysfunction as per elevations in liver transaminases (AST, ALT), serum ALP, and GGT enzymes, in addition to a significant decrease in the total protein concentrations. These findings could be attributed to the oxidative stress that was mediated by DOX injection, which attacked hepatocytes and consequently released hepatic enzymes from the damaged cells to the blood serum. They also might be due to alterations in protein synthesis and/or metabolic functions of hepatocytes. Decreasing the hepatic toxicity upon AVN-C treatment indicates that the AVNS has a therapeutic effect against liver dysfunction and cellular injury of the liver that were induced by DOX. Our data was in agreement with Tousson et al. (2022), who reported the potential curative role of AVNs against oxidative stress induced by acute hepatotoxicity in rats.

The present study found that DOX treatment resulted in elevation in MDA and depletions in GSH, SOD, and CAT in liver homogenates, consistent with previous studies demonstrating the effects of DOX on the imbalance of hepatic oxidants/antioxidants (Sirwi et al., 2021; Saleh et al., 2022; El-Said K. S. et al., 2023). Treating DOX-challenged rats with AVN-C significantly improved the reversal of changes in hepatic antioxidant/oxidant hemostasis of the DOX-injected rats, as it can inhibit lipid peroxidation and prevent oxidative stress. This suggested that AVN-C acts on DOX-promoted hepatotoxicity through its antioxidant properties, potentially by enhancing the antioxidant defense system in accordance with previous studies demonstrated the cytoprotective effects of AVN-C against oxidative damage and enhancement of antioxidant status (Wang and Eskiw, 2019). In the current investigation, the beneficial hepatoprotective effects of AVN-C could be due to the anti-inflammatory properties of AVN-C; it was found to reduce the key inflammatory mediators associated with DOX-induced liver injury. The current findings concur with previous reports that highlighted the potent *in vivo* anti-inflammatory efficacy of AVNs (Zhang et al., 2020; Tousson et al., 2022; Wankhede et al., 2023). A previous study reported that herbal compounds mitigated DOX-induced liver toxicity in rats by countering oxidative stress and inflammation (Ahmed et al., 2022). Our results revealed that AVN-C exert pronounced pharmacological actions as antioxidant, and anti-inflammatory, which indicated that AVS is a prospective therapeutic agent against DOX-induced toxicity.

The phosphorylated Akt triggers the activation of multiple downstream proteins that mediate signals encouraging cell proliferation, differentiation, and death. GSK-3β may be phosphorylated and its activity inhibited by the activated Akt (Saleh et al., 2022). Interestingly, the present study showed that GSK-3β inhibition provides guarding against DOX-prompted hepatic tissue damage accompanied by elevation of the Akt at the gene and protein expression levels. In accordance with a previous study by Abbas and Kabil (2017), who reported that liraglutide ameliorates DOX toxicity through the Akt/GSK-3β signaling pathway in rats. It has been reported that inhibition of the Wnt-4/β-catenin signaling pathway could be potential therapeutic targets in liver disorders. Our investigation showed the hepatoprotective effect of AVN-C was associated with the downregulation of the Wnt-4 and β-catenin expressions. Herein, attenuation of hepatotoxicity by DOX treatment through inhibition of GSK-3β and Wnt-4/β-Catenin might be a promising strategy of AVN-C to counteract DOX-induced hepatotoxicity. These findings were in line with previous studies that established the inhibition of the Wnt-4/β-Catenin signaling pathway by natural compounds in experimental animals (Li et al., 2014; Cao et al., 2022; Bakrania et al., 2023). Furthermore, a previous study reported the activation of WNT/β-catenin signaling pathway is involved in the pathogenesis of CCl_4_-induced liver fibrosis and protective role of natural compounds against liver fibrosis by inhibiting the Wnt/β-catenin pathway (El-Ashmawy et al., 2020). Consistently, our histopathological investigations evidenced biochemical analysis indicating severe hepatocellular damages by DOX injection in rats. These results are consistent with recent findings about DOX-induced hepatotoxicity (AlAsmari et al., 2021; Saleh et al., 2022; El-Said K. S. et al., 2023). By significantly reducing central venous congestion, cell infiltration, degeneration, the number of hepatocytes with pyknotic, vacuolization, and sinusoidal narrowing, AVN-C treatment dramatically restores the histological abnormalities and increased hepatic enzyme activity seen in DOX-treated rats, suggesting protection against DOX-induced liver injury. A previous study reported that taurine protects DOX-induced hepatotoxicity via its membrane-stabilizing effect and improvement of histopathological changes in rats (Gedikli et al., 2023).

## 5 Conclusion

**FIGURE 10:**
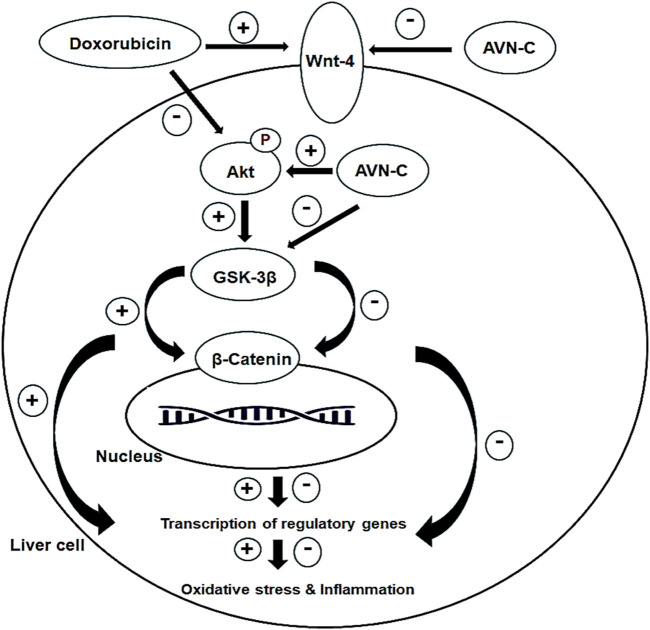
Diagram shows the effect of AVN-C against DOX-induced hepatotoxicity through inhibiting oxidative stress, inflammation, and modulating the Akt/GSK-3β and Wnt-4/β-Catenin pathways.

This study is the first to demonstrate that AVN-C has a significant attenuative effect against DOX-induced hepatotoxicity in male rats. This is achieved by enhancing the liver’s antioxidant capacity, inhibiting hepatic inflammatory cytokines, and modulating the Akt/GSK-3β and Wnt-4/β-Catenin pathways (Figure 10). The combination of AVN-C and DOX shows promise and effective chemotherapy strategy. Further research should explore the potential of AVN-C in ameliorating other toxicities induced by DOX and investigate the underlying mechanisms.

## Data Availability

The original contributions presented in the study are included in the article/Supplementary Materials, further inquiries can be directed to the corresponding authors.
