# Corrigendum: Avenanthramide-C ameliorate doxorubicin-induced hepatotoxicity via modulating Akt/GSK-3β and Wnt-4/β-Catenin pathways in male rats

**DOI:** 10.3389/fmolb.2025.1601841

**Published:** 2025-05-09

**Authors:** Maha Abdullah Alwaili, Amal S. Abu-Almakarem, Salwa Aljohani, Sahar Abdulrahman Alkhodair, Maha M. Al-Bazi, Thamir M. Eid, Jehan Alamri, Maysa A. Mobasher, Norah K. Algarzae, Arwa Ishaq A. Khayyat, Luluah Saleh Alshaygy, Karim Samy El-Said

**Affiliations:** ^1^ Department of Biology, College of Science, Princess Nourah bint Abdulrahman University, Riyadh, Saudi Arabia; ^2^ Department of Basic Medical Sciences, Faculty of Applied Medical Sciences, Al-Baha University, Al Bahah, Saudi Arabia; ^3^ Chemistry Department, Faculty of Science, Taibah University, Yanbu, Saudi Arabia; ^4^ Department of Biochemistry, Faculty of Science, King Abdulaziz University, Jeddah, Saudi Arabia; ^5^ Biology Department, Faculty of Science, King Abdulaziz University, Jeddah, Saudi Arabia; ^6^ Department of Pathology, Biochemistry Division, College of Medicine, Jouf University, Sakaka, Saudi Arabia; ^7^ Department of Physiology, College of Medicine, King Saud University, Riyadh, Saudi Arabia; ^8^ Biochemistry Department, Science College, King Saud University, Riyadh, Saudi Arabia; ^9^ Biochemistry Division, Chemistry Department, Faculty of Science, Tanta University, Tanta, Egypt

**Keywords:** avenanthramides, antioxidants, anti-inflammatory, doxorubicin, hepatotoxicity, signaling pathway

In the published article, an **Author Name** was incorrectly written as Norah K. Algarza. The correct spelling is Norah K. Algarzae.

The authors apologize for this error and state that this does not change the scientific conclusions of the article in any way. The original article has been updated.

